# Regional Wind Variability Modulates the Southern Ocean Carbon Sink

**DOI:** 10.1038/s41598-019-43826-y

**Published:** 2019-05-14

**Authors:** Lydia Keppler, Peter Landschützer

**Affiliations:** 1 Max-Planck-Institute for Meteorology (MPI-M), Hamburg, Germany; 2 International Max Planck Research School on Earth System Modelling (IMPRS-ESM), Hamburg, Germany

**Keywords:** Marine chemistry, Physical oceanography

## Abstract

The Southern Ocean south of 35°S accounts for approximately half of the annual oceanic carbon uptake, thereby substantially mitigating the effects of anthropogenic carbon dioxide (CO_2_) emissions. The intensity of this important carbon sink varies considerably on inter-annual to decadal timescales. However, the drivers of this variability are still debated, challenging our ability to accurately predict the future role of the Southern Ocean in absorbing atmospheric carbon. Analysing mapped sea-air CO_2_ fluxes, estimated from upscaled surface ocean CO_2_ measurements, we find that the overall Southern Ocean carbon sink has weakened since ~2011, reversing the trend of the reinvigoration period of the 2000s. Although we find significant regional positive and negative responses of the Southern Ocean carbon uptake to changes in the Southern Annular Mode (SAM) over the past 35 years, the net effect of the SAM on the Southern Ocean carbon sink variability is approximately zero, due to the opposing effects of enhanced outgassing in upwelling regions and enhanced carbon uptake elsewhere. Instead, regional shifts in sea level pressure, linked to zonal wavenumber 3 (ZW3) and related changes in surface winds substantially contribute to the inter-annual to decadal variability of the Southern Ocean carbon sink.

## Introduction

The global oceans absorb ~25% of the annually emitted carbon dioxide (CO_2_) from human activities^1^. A disproportionally large part of this uptake is linked to the Southern Ocean south of 35°S, which accounts for ~50% of the annual oceanic CO_2_ uptake^2^ and where ~40% of the anthropogenic CO_2_ since the beginning of industrialisation is stored^3–5^. Therefore, the Southern Ocean plays a substantial role in mitigating the effects of human carbon emissions and understanding this carbon sink and its related processes is crucial for future climate projections.

A sobering study by Le Quéré *et al*.^6^ showed that despite the continued increase in atmospheric CO_2_, the Southern Ocean carbon sink saturated in the 1990s, diverging from the expected uptake based on thermodynamic considerations. The authors explained this saturation with a positive trend in the Southern Annular Mode (SAM), i.e., the dominant mode of variability in the Southern Ocean, describing the zonal pressure difference between 40°S and 65°S^7^. This positive trend led to an intensification and poleward shift of the westerly winds, the driving force behind the Southern Ocean upwelling of carbon-rich deep water^7–11^. The link between the saturation of the Southern Ocean carbon sink in the 1990s and the positive SAM phase was later confirmed by other model and atmospheric inverse studies^12–16^.

Further studies have demonstrated that the response of the mixed-layer depth and temperature to the SAM is not as “annular” (ring-shaped) as previously thought, and is in fact zonally asymmetric, possibly affecting the Southern Ocean carbon uptake^17,18^ (see also S1). Due to the scarcity of observational data, many previous studies focused on zonal averages of the whole Southern Ocean. Although this view has helped to understand the mean dynamics in the last two decades, it is becoming more and more evident that the Southern Ocean is not zonally uniform and that many key processes occur in different regions that are averaged out in zonal averages^19,20^.

Recent technical advancements and efforts by the scientific community have led to basin-wide observation-based estimates of the sea-air CO_2_ flux, sea surface temperature (SST), and sea surface salinity (SSS). To overcome the paucity of CO_2_ measurements, novel approaches based on statistical relationships and machine-learning algorithms have advanced our ability to extrapolate and basin-wide map the information collected from single sampling routes^21^.

Using the mapped partial pressure of CO_2_ (pCO_2_) data until December 2011, a study established that the saturation trend of the 1990s stopped and reversed between the early 2000s and 2011 and that the Southern Ocean had returned to its expected uptake strength^22^. Despite the shipboard-based pCO_2_ estimates being heavily extrapolated, longer-term signals, such as the decadal fluctuations that mark the saturation and reinvigoration periods were identified as robust features among different approaches^23,24^, and the reinvigoration of the Southern Ocean carbon sink was later confirmed by several other studies^23,25,26^.

Despite increasing evidence for the strengthening of the Southern Ocean carbon sink in the 2000s, the processes behind this strengthening are still debated, and the future evolution of this important sink region is highly uncertain. One proposed mechanism is a zonally asymmetric atmospheric circulation, which led to an oceanic dipole of warming and cooling that in turn increased the CO_2_ uptake during the Southern Ocean reinvigoration period (2002 through 2011)^22^. Another explanation is based on changes in the upper meridional overturning circulation (MOC), which may be linked to trends in the SAM^25^. Another study argues that the inter-annual drivers of the Southern Ocean carbon sink are seasonally decoupled, with wind stress as the main driver in austral winter and biology in austral summer^26^.

Here, we build on previous assessments using neural-network derived mapped pCO_2_ estimates based on shipboard measurements to demonstrate the temporal evolution of the Southern Ocean carbon sink and its regional drivers. We focus on the period after the end of the reinvigoration in 2011 and put our findings from this most recent period in context with previous findings since the 1980s.

## Results and Discussion

### The Southern Ocean carbon sink variability

**Figure 1:**
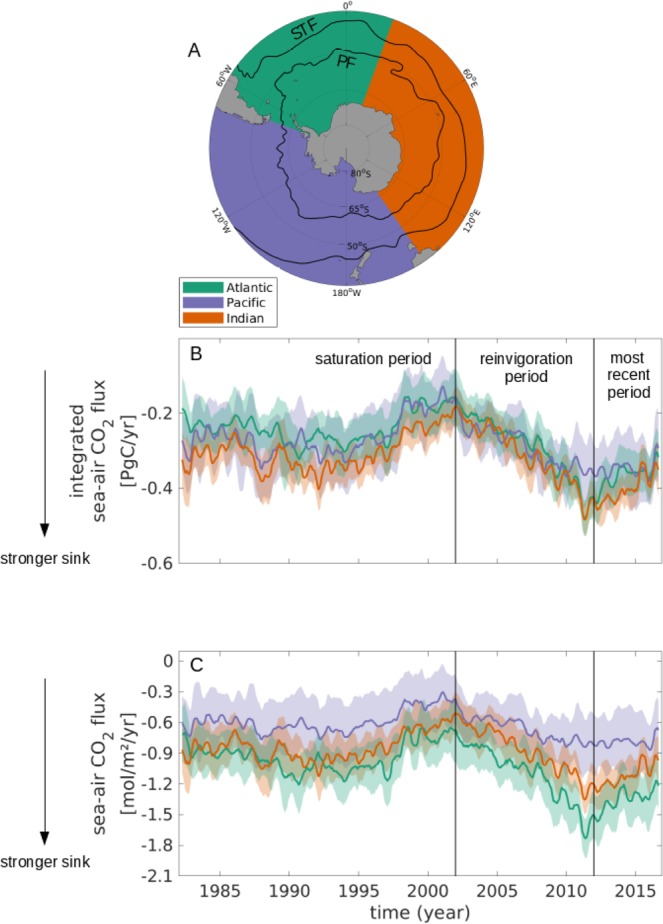
The evolution of the Southern Ocean Carbon sink by sectors between 35°S and the Antarctic coast from 1982 through 2016. (**A**) The sectors and fronts in the Southern Ocean, illustrating the Atlantic (green), Pacific (purple), and Indian (orange) sectors in color, and the Subtropical Front (STF) and Polar Front (PF) from Orsi *et al*.^50,52^ as black solid lines. (**B**) Timeline of the integrated sea-air carbon flux in the Atlantic (green), Pacific (purple), and Indian (orange) sectors. (**C**) Timeline of the sea-air carbon flux per unit area in the Atlantic (green), Pacific (purple), and Indian (orange) sectors.

Using an updated observation-based mapped estimate of the sea-air CO_2_ flux (extended from Landschützer *et al*.^2^), we find that the substantial decadal variability of the Southern Ocean carbon sink persists and is present in all three sectors: the reinvigoration period of increased CO_2_ uptake lasted until ~2011, and is followed by a reversal of this trend with decreasing carbon uptake until the end of our study period in December 2016 (Fig. 1B,C), consistent with a previous finding^26^.

The integrated CO_2_ uptake (Fig. 1B) does not differ considerably between the three sectors despite the large differences in area (Atlantic sector: ~2.2 * 10^7^ km^2^, Pacific sector: ~3.7 * 10^7^ km^2^, and Indian sector: ~3.0 * 10^7^ km^2^, Fig. 1A). Specifically, the integrated sea-air CO_2_ flux from 2012 through 2016 is approximately equal in each of the three sectors with a mean uptake of 0.3 to 0.4 PgC yr^−1^ resulting in a total Southern Ocean carbon uptake of ~1.1 ± 0.2 PgC yr^−1^, or approx. 50% of the contemporary annual mean oceanic carbon uptake. The comparable uptake strength between sectors is in agreement with previous results, who found a fairly homogeneous carbon uptake between the three sectors from different model and inversion estimates^27^.

Despite the sectoral similarities in the integrated CO_2_ uptake, strong sectoral differences exist in the magnitude of the sea-air CO_2_ flux per unit area (Fig. 1C). In particular, the Atlantic sector, i.e., the sector with the smallest spatial extent, reveals the largest variability range from ~−0.7 mol m^−2^ yr^−1^ in the early 2000s to ~−1.7 mol m^−2^ yr^−1^ in 2011. Throughout most of the time period, the Atlantic sector is the most intense carbon sink per unit area within the Southern Ocean and from 2012 onward, the CO_2_ uptake per unit area in the Atlantic sector (~1.4 mol m^−2^ yr^−1^) is nearly twice the amount taken up by the Pacific sector (~0.8 mol m^−2^ yr^−1^) and still considerably more than in the Indian sector (~1.1 mol m^−2^ yr^−1^). This strong mean uptake has been recently challenged using calculated pCO_2_ from biogeochemical Argo floats^28,29^. While the differences are not yet fully resolved, a combination of float and ship data as a next step is required to fully constrain both the seasonal cycle and the mean uptake in the Southern Ocean. We therefore focus on the inter-annual variability and regional differences rather than the integrated carbon uptake in this study.

Another striking observation is that since the late 2000s, stronger differences between the sectors emerge. In the saturation period of the 1990s and the following reinvigoration period in the early 2000s, differences between the sectors stay within one standard deviation around the mean, and they agree on the direction of the trend. However, since approx. 2008, the sink strength in the Pacific sector stalls, whereas the Atlantic and the Indian sectors continue to take up additional carbon until ~2011, followed by a sink reduction thereafter, causing a significant divergence in the uptake intensity between the Atlantic and Pacific sectors.

It is a possibility that the sectoral differences towards the end of the time line are partially due to increased observational data in these years. This is however challenging to test with the available measurements, and model-based observing system simulations might be required to address this question.

### The SAM’s effect on the Southern Ocean carbon sink

The SAM, the dominant climate mode of variability in the Southern Ocean, influences the MOC, and hence the uptake and outgassing of carbon^9–11^. Specifically, in positive SAM phases, the westerly winds in the Southern Ocean intensify and shift poleward^11^. This intensification leads to enhanced Ekman transport, resulting in an increase in both upwelling and subduction, and hence outgassing and uptake, respectively^6,13,30^.

**Figure 2:**
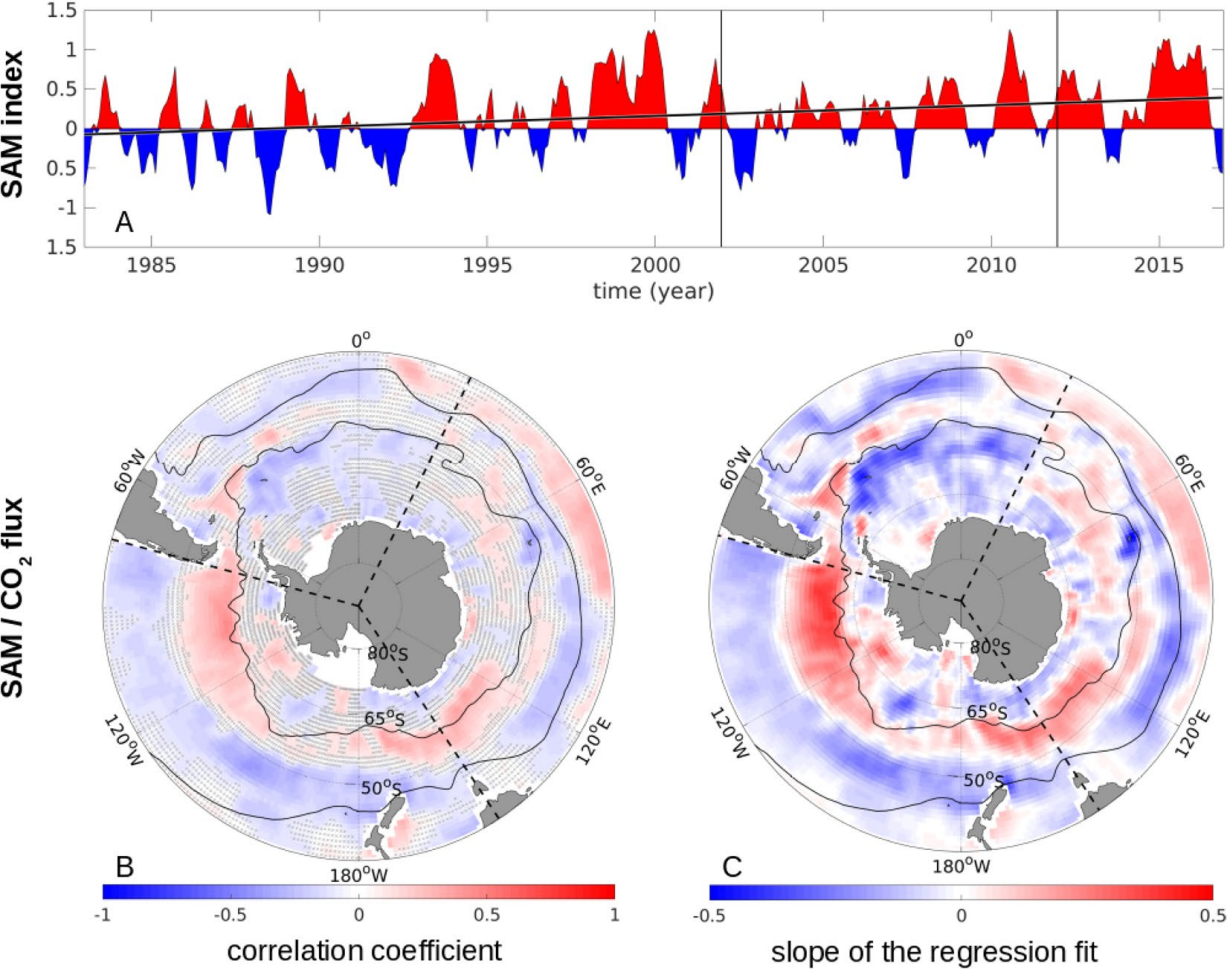
The relationship between the SAM index and the CO_2_ flux anomaly from January 1982 through 2016. (**A**) Standardized SAM index, smoothed with a 3-month running mean, and the trend line in black. Positive SAM indices are illustrated in red, negative ones in blue. The start of the reinvigoration (Jan 2002) and the most recent period (Jan 2012) are marked with thin vertical black lines. (**B**) The correlation coefficients between the sea-air CO_2_ flux anomaly [mol m^−2^ yr^−1^] and the smoothed, standardized SAM index. Coefficients with significance <95% are hatched. (**C**) The slope of the regression fit between the sea-air CO_2_-flux anomalies [mol m^−2^ yr^−1^] and the standardized SAM index. As the SAM index is standardized to have a mean of 0 and a standard deviation of 1, (**C**) illustrates the change in the CO_2_ flux [mol m^−2^ yr^−1^] per standard deviation of the SAM. (**B**,**C**) The mean positions of the PF and the STF are illustrated as thin black lines, the three Southern Ocean sectors are delimited by dashed black lines, and coastal areas are masked white.

A positive trend in the SAM index polarity was suggested as the driver behind the Southern Ocean carbon sink stagnation in the 1990s^6^. Similarly, a more recent study found that in a region south of Tasmania, there are regions of both increased carbon uptake and outgassing in positive SAM phases in austral summer^31^. When considering the period from 1982 through 2016, the SAM index illustrates substantial variations in time; however, it further shows a continuous positive long-term trend (Fig. 2A). Therefore, we first investigate if the SAM affects the Southern Ocean carbon sink as a whole when considering the entire 35-year period (1982 through 2016). A 2D correlation and regression analysis confirms the link between the SAM and the carbon uptake but highlights the contrasting regional differences within the Southern Ocean (Fig. 2). The resulting pattern closely reflects the results of a model-based study^13^.

In agreement with that study^13^, positive SAM phases correlate with anomalous outgassing in the region between ~50°S and ~65°S, with the exception of the Atlantic sector (Fig. 2B), potentially illustrating the recently suggested zonal SAM asymmetry^17,18^. However, we find that for most of the remaining Southern Ocean, the CO_2_ flux correlates negatively with the SAM index; here, positive SAM phases are linked to increased uptake. The general picture is comprised of alternating zonal bands with positive and negative correlations. However, the pattern in the Atlantic sector is approximately opposite to the Pacific sector south of ~45°S,

Regionally, the link between the SAM and the air-sea exchange of CO_2_ derived from mapped shipboard observations is evident. Just north of the PF in the Pacific sector, anomalous outgassing of approx. 0.5 mol m^−2^ yr^−1^ occurs per standard deviation of the SAM (Fig. 2C). Conversely, south of the PF in the Atlantic sector, anomalous carbon uptake of ~0.4 mol m^−2^ yr^−1^ occurs per standard deviation of the SAM.

However, when integrating the total effect of the SAM on the Southern Ocean carbon uptake south of 35°S, we find that the regionally opposing effects cancel each other out: the net effect is 0.0 PgC yr^−1^ per standard deviation of the SAM, for the whole Southern Ocean, and the net effect in each of the three sectors is also 0.0 PgC yr^−1^. Inversion and model-based studies have also found a compensation of positive and negative correlations between the sea-air CO_2_ flux and the SAM throughout the Southern Ocean^12–14^. These studies found a slightly positive net integrated uptake of ~0.1 PgC yr^−1^ per standard deviation of the SAM in their study periods. However, our findings based on upscaled observations suggest that the positive trend in the SAM does not considerably alter the basin-wide net Southern Ocean CO_2_ uptake over the past 35 years.

### Physical sea surface properties and the carbon flux from 2012 through 2016

Despite its regional correspondence and its link to the saturation of the Southern Ocean carbon sink in the 1990s^6^, the SAM index polarity does not fully explain the overall Southern Ocean carbon sink variability over the 35-year period. We therefore continue to investigate other potential drivers.

**Figure 3:**
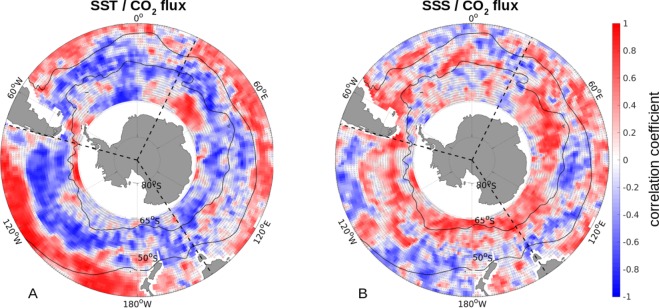
The correlation coefficients between the sea-air CO_2_ flux anomaly (negative is into the ocean) and SST (**A**) and SSS (**B**) anomalies from 2012 through 2016. The trend and seasonal cycle was removed from all three variables, and then smoothed with a 3-month running average. The mean positions of the PF and the STF are illustrated as thin black lines, the three Southern Ocean sectors are delimited by dashed black lines, and coastal areas are masked white. Coefficients with significance <95% are hatched.

As CO_2_ is more soluble in colder water, one would expect positive correlations between SST and sea-air CO_2_ flux anomalies in regions where the solubility of CO_2_ is the dominant driver (negative SST anomalies corresponding to negative sea-air CO_2_ flux anomalies). Instead, the general picture during this period are alternating zonal bands of positive and negative correlations. Specifically, warmer SST correspond to less uptake in the northern region of subduction, to less outgassing in the upwelling band, i.e., where circulation and/or biology dominate the CO_2_ flux variability^21,32^, and patches of less uptake in the southern regions of deep water formation (Fig. 3A, see also S4–S6).

Hence, in the northern zonal band (north of ~40°S) solubility drives the CO_2_ flux variability. In contrast, south of 40°S the band of negative correlations suggests other processes to be dominant, such as variations in dissolved inorganic carbon (DIC) and alkalinity^33^. This zonal symmetry suggests different drivers than explored in the reinvigoration period, where the authors found that in the Pacific Sector of the Southern Ocean changes in the thermal component dominated over the non-thermal counterpart^22^.

In contrast, the correlation between SSS and CO_2_ flux anomalies reveals only some significant patches at the 95% confidence level (Fig. 3B).

### Regional shifts in sea level pressure (SLP) and surface winds as CO_2_ flux drivers

As we have demonstrated in the previous section, changes in the non-thermal drivers (i.e. DIC, alkalinity or biology), and not solubility, are the dominant processes behind the recent Southern Ocean carbon sink. Although the atmospheric forcing on the ocean dynamics is generally non-linear^34^, the relationship between atmospheric forcing and ocean dynamics has been suggested in the past to influence the Southern Ocean carbon uptake^6,25^. Here, we demonstrate that regional shifts in SLP and the related winds affect the MOC, modulating the Southern Ocean carbon sink.

The southern extra-tropical atmospheric circulation is overall zonally symmetric, but significant asymmetries, such as zonal wavenumbers 1 and 3 (ZW1 and ZW3, respectively) are present within this zonal flow^35,36^. ZW1 and ZW3 are quasi-stationary, where ZW1 is a zonal wave with one ridge in the Pacific sector and one trough in the Atlantic sector, while ZW3 has ridges south of each of the three continents and three troughs in between^36,37^. The observed picture is generally a combination of both ZW1 and ZW3, while ZW1 tends to be considerably more dominant^36,38^.

From 2002 through 2011, a more zonally asymmetric atmospheric circulation was suggested to lead to an oceanic dipole of warming and cooling, which was identified to drive the reinvigoration of the Southern Ocean carbon sink (22, see also S7). Due to geostrophic balance, the winds follow this pattern, resulting in stronger zonal winds in the Pacific sector, and weaker zonal winds in the Atlantic and Indian sectors. In turn, anomalous northward advection in the Pacific sector led to enhanced upwelling of cold water, enabling enhanced carbon uptake due to the solubility of CO_2._ Concurrently, anomalous southward advection in the Atlantic sector led to enhanced downwelling and carbon uptake in that area^22,39^. The SLP in this time period resembles the inverse structure of the typical ZW1 pattern^22^ (see also S7) with an additional imprint of the ZW3 pattern^39^.

**Figure 4:**
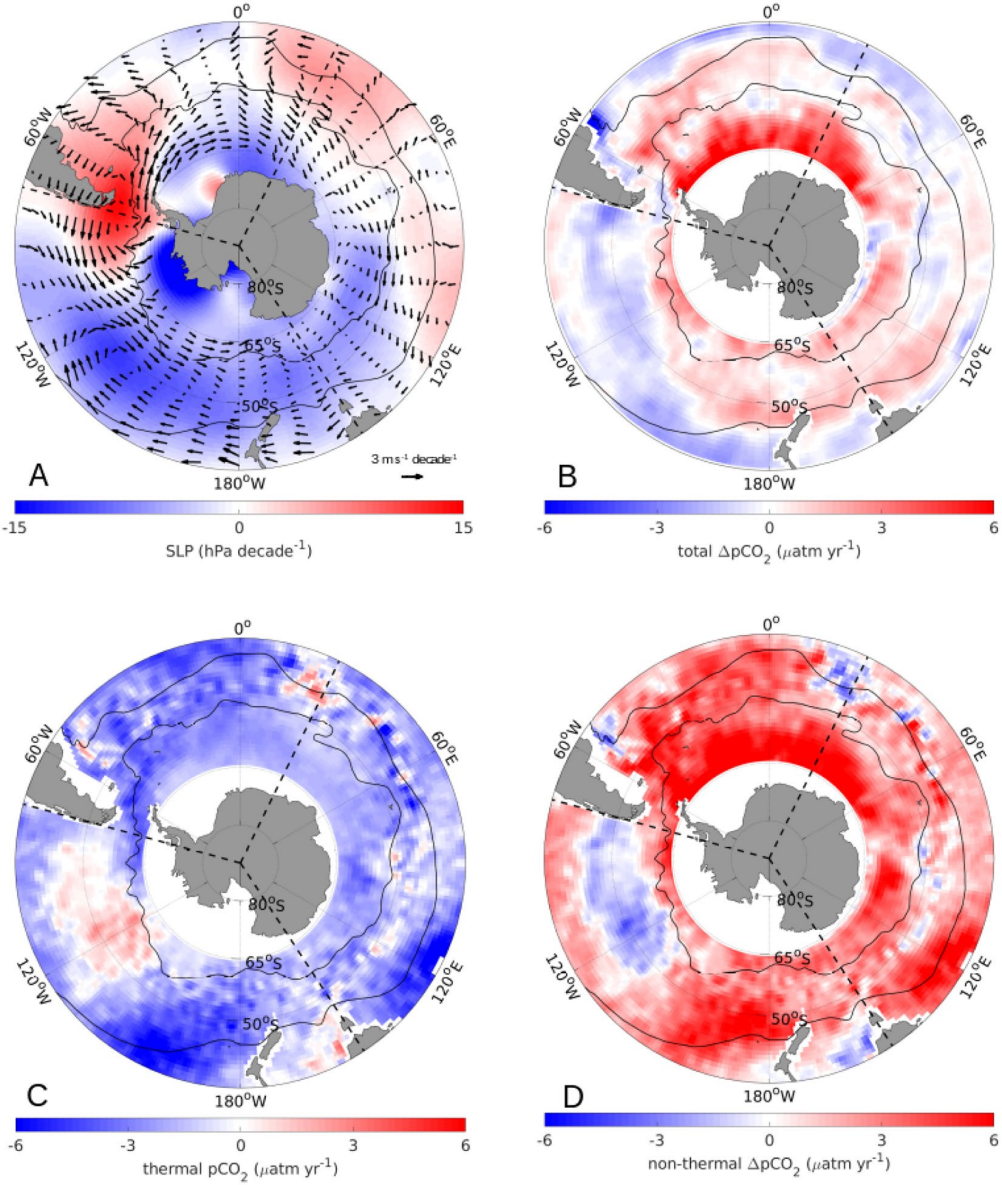
Trends of SLP and 10 m wind velocity and the trends of the ΔpCO_2_, (oceanic pCO_2_ – atmospheric pCO_2_) and its components during the most recent period (2012 through 2016). (**A**) Trend of the SLP (hPa decade^−1^) (color) and trend of the 10 m wind velocity [m s-1 decade^−1^] (vectors). (**B**) Trend of the ΔpCO_2_ (μatm year^−1^); (**C**) trend of the thermal component of the pCO_2_ (μatm year^−1^); (**D**) trend of the non-thermal component of the ΔpCO_2_ (μatm year^−1^). The mean positions of the PF (~55°S) and the STF (~40°S) are illustrated as thin black lines, the three Southern Ocean sectors are delimited by dashed black lines. See S7 for the analogous figure for the reinvigoration period (2002 through 2011).

Based on this finding, it appears plausible that a dominance shift of ZW1 or ZW3 might drive the most recent Southern Ocean carbon sink stagnation. Indeed, from 2012 through 2016, the trends in SLP and resulting surface wind velocity have shifted substantially again compared to both the saturation and reinvigoration periods (Fig. 4A; see also S7). In this period, we find a strong asymmetry in the local pressure system with a positive SLP trend over the Drake Passage (~30°W), south of Africa (~20°E), and west of Australia (~100°E), and negative SLP trends in between (Fig. 4A). This pattern strongly resembles the positive ZW3 pattern^36^, with the exception that typical ZW3 patterns are more symmetric, with the third ridge being further east, just south of Australia^36,37^. This is in line with a recent study by Schlosser *et al*.^40^, who found that 2016 has a strong positive phase in the ZW3, causing significant decay of Antarctic sea ice.

Due to geostrophic balance, winds tend to follow the SLP gradient, as seen in Fig. 4A. We find that south of the PF in the Atlantic and Indian sectors, the local trends enhance the westerly wind circulation, while at the same latitudes in the eastern Pacific the local trends counteract the westerly circulation.

Previous studies have shown that enhanced westerlies enhance the MOC, while decreased westerlies decrease the MOC^6,25^. To investigate the effects of the changes in the MOC, we consider the changes in pCO_2_. The ΔpCO_2_ (oceanic pCO_2_ – atmospheric pCO_2_) trends from 2012 through 2016 are predominantly positive south of the PF (Fig. 4B), indicating reduced uptake close to the seasonally ice-covered regions. In addition, the total ΔpCO_2_ has mainly a negative trend north of the STF in all three sectors, while between the PF and the STF, the trends are mainly positive (i.e., reduced uptake/increased outgassing), with the most dominant exception being the eastern Pacific sector around 50°S. The recent decrease in the carbon uptake per unit area in the Atlantic and Indian sectors shown in Fig. 1C is hence mainly due to a decrease in carbon uptake in the higher latitudes, which is slightly offset by the increased uptake north of the STF. Similarly, the recent stagnation in the carbon uptake per unit area in the Pacific sector is largely due to increased uptake towards the north-eastern Pacific sector being offset by decreased uptake towards the south-western Pacific sector.

To determine the processes behind the trends in the total ΔpCO_2_, we further separate the observed trends in the surface ocean pCO_2_, using the CO_2_ sensitivity of seawater to thermal changes of 4.23%/°C^22,32^. As CO_2_ dissolves faster in colder water, areas with negative trends in the thermal component of pCO_2_ are regions that enhance the carbon uptake^41^. The trend in the thermal component (Fig. 4C) is mainly negative, i.e., surface waters cooled over the past few years, with a few exceptions, most notably in the eastern Pacific sector north of the PF, thereby enhancing the solubility of seawater.

The non-thermal component is comprised of the sum of circulation and biological effects. Regions of upwelling are usually associated with outgassing, while subduction areas tend to be regions of carbon uptake. Moreover, regions of high biological productivity tend to be regions of carbon sequestration. The pattern of the trend of the non-thermal component of the ΔpCO_2_ (Fig. 4D) closely resembles the pattern of the trend of the total ΔpCO_2_ (Fig. 4B), with the thermal component offsetting the non-thermal component.

Combining the findings from Fig. 4, we find that in the Atlantic and Indian sectors, south of the STF, increased winds enhance the westerly circulation (Fig. 4A), likely resulting in an increase in Ekman-induced upwelling of carbon-rich waters from deeper layers, which explain the observed anomalous outgassing and northward transport of cold and carbon-rich waters in these two sectors south of the STF (see Fig. 4B,D). In contrast, at the same latitudes in the Pacific sector, decreased winds as a result of the high-pressure area at Drake Passage explain the observed decreased carbon uptake and decreased outgassing here, likely imposed by reduced upwelling and subduction. In contrast, the inflow of warmer surface waters from the north, induced by enhanced westerlies, only in part counteracts the non-thermal signature. Concurrently, Fig. 4A reveals enhanced winds in the west of the Pacific sector leading to enhanced upwelling and subduction, and hence both increased carbon uptake and increased outgassing. These opposing effects lead to the overall CO_2_ flux stagnation of the Pacific sector in this period.

Our finding that the carbon uptake in the Pacific sector is mainly driven by the non-thermal component, is somewhat contrary to previous findings that trends in this region are solubility driven^22^, but might also indicate that the relative dominance between thermal and non-thermal drivers is shifting in time, highlighting the complexity of the Southern Ocean carbon sink.

## Summary and Conclusions

In summary, our study demonstrates that regionally, the Southern Ocean carbon uptake shows a significant regional correspondence to the SAM index polarity, although when considering the entire 35-year period, the SAM does not have a considerable effect on the overall Southern Ocean carbon uptake. Instead, regional shifts in SLP closely tied to the ZW3 pattern in the Southern Ocean and related surface wind velocity substantially affect the most recent evolution of the Southern Ocean carbon sink. In the Atlantic and Indian sectors, enhanced outgassing in upwelling regions and decreased uptake in subduction regions dominate after 2011, causing the carbon sink in these sectors to weaken. In the Pacific sector, however, regionally opposing trends to the east and the west linked to the ZW3 pressure asymmetry cause the net carbon sink of this sector to stall. In particular, towards the eastern Pacific sector, local wind patterns counteracting the mean westerly flow lead to decreased upwelling of carbon from deeper ocean layers, while towards the west, local winds enhancing the westerly flow lead to enhanced stirring and outgassing of carbon. Our results also reveal a rather complex picture of the Southern Ocean carbon sink. While from 2002 through 2011 it was suggested that the increase in solubility led to more carbon uptake in the Pacific sector^22^, in the subsequent years the wind-driven upward stirring caused a slow-down of the uptake in the eastern part of this basin. Our findings therefore suggest that the evolution of the Southern Ocean carbon sink is not only determined by local weather patterns but further determined by the relative dominance of thermal and non-thermal drivers that appear to locally interchange dominance in time.

Our study implies that adequate observations of SLP and winds in the Southern Ocean are key to better understand the regional processes in this dynamic region on inter-annual to decadal timescales. Similarly, future studies including better representation of regional weather patterns in earth system models may lead to a better modelled representation of the Southern Ocean carbon cycle and close the present discrepancies between model-based and observation-based sea-air fluxes^39^.

It is an open question of how the Southern Ocean carbon sink will continue to evolve. However, we demonstrate that understanding the evolution of regional weather patterns is key in monitoring the Southern Ocean sink strength on inter-annual to decadal timescales.

## Data and Methods

We combine data from different platforms in the Southern Ocean south of 35°S, which we introduce below.

### Ship-based sea-air CO_2_-flux estimate

We use a neural-network derived mapped estimate of the sea-air CO_2_ flux, which is based on data from the Surface Ocean CO_2_ Atlas database SOCATv5^42^. To overcome the paucity of shipboard pCO_2_ observations, this product applies a 2-step neural-network mapping approach, using a suite of independent predictors as proxy data to infer the final pCO_2_ fields. In the first step of this SOM-FFN method, a self-organizing map (SOM) clusters the global ocean into biogeochemical provinces. In the second step, a feed-forward network (FFN) is applied to determine the statistical relationships between the SOCATv5 data^42^ and proxy parameters within the provinces to then estimate the pCO_2_. Lastly, the sea-air CO_2_ flux is computed using a bulk flux formulation, where positive values indicate outgassing, and negative values indicate oceanic uptake of CO_2_. The gas transfer is computed using a quadratic wind dependence^43^ based on ERA-interim wind speeds^44^. The gas transfer coefficient is then scaled so that the mean transfer velocity of 16 cm hr^−1^ matches a recent estimate by Wanninkhof *et al*.^45^ For more information on this method see Landschützer *et al*.^46^ and for a discussion on the robustness of this data estimate, see S2. This mapped estimate is on a 1° × 1° monthly grid, originally created from 1982 through 2011. We extend it by five additional years until December 2016. We compute the CO_2_ flux anomalies by removing the climatological seasonal cycle and smooth the remaining high-frequency variability using a 3-month running mean.

### The SAM index

We use the SAM index by Marshall *et al*.^7^, which is based on the observed pressure difference between six stations at 40°S and 65°S. We standardise the index by subtracting the mean and dividing it by the standard deviation over the time period (1958 to 2017), following Lovenduski *et al*.^13^. We then smooth the standardised index with a 3-month running mean in order to be able to analyse the inter-annual signal of the SAM, following Lenton and Matear^12^. Although some studies do not smooth the SAM index at all, others smooth with a running mean of 8 or 12 months^12–14^. We tested different high-pass and low-pass filters and found that the 3-month running mean can represent the inter-annual variability of the SAM index without removing too much of the signal.

### Argo float-based SST and SSS

Argo floats are autonomous profiling floats that measure seawater properties in the water column (http://www.argo.ucsd.edu/). As such, they fill large observational gaps in the ocean, especially in historically under-sampled regions, such as the Southern Ocean. The Roemmich and Gilson^47^ Argo-based product provides optimally interpolated data of temperature and salinity of the top 2000 m on a monthly 1° × 1° grid. Due to the relatively high spatiotemporal density of floats compared to ship data, this data set is of high confidence and provides reliable *in-situ* data. We use the shallowest value at 2.5 m of the temperature and salinity for the SST and SSS respectively from January 2004 until December 2016 (i.e., 13 years of data). Based on the data availability of this product, the analysis of the sea surface properties only extends until 65°S. It would be interesting to analyse the region south of 65°S as well, as this is a region of deep water formation and hence subduction. However, as this region is partially ice-covered, there are few Argo profiles with good quality control flags, which results in data with lower confidence than the gridded Argo-based data we use in this study. It has to be left for future analyses to investigate the relationship between the physical sea surface properties and the carbon sink in this region. As for the sea-air CO_2_ flux, we compute anomalies by removing the climatological seasonal cycle and we smooth remaining high-frequency variability using a 3-month running mean.

### SLP and surface wind velocity

To analyse how the SLP and related wind velocity affects the Southern Ocean carbon uptake, we use reanalysis data between January 2004 and December 2016. For the SLP, we used the NCEP/NCAR Reanalysis monthly mean data (/www.esrl.noaa.gov/psd/data/gridded/data.ncep.reanalysis.surface.html), and for the wind velocity, we use the monthly mean zonal and meridional 10 m wind velocity components from Era Interim (http://apps.ecmwf.int/datasets/data/interim-full-daily/levtype=sfc/).

### Separation into thermal and non-thermal components and ΔpCO_2_

Following Takahashi *et al*.^32^, we separate the thermal and non-thermal components of the pCO_2_ at each grid point using equation (1) and (2):

1 $${\rm{n}}{\rm{o}}{\rm{n}}-{\rm{t}}{\rm{h}}{\rm{e}}{\rm{r}}{\rm{m}}{\rm{a}}{\rm{l}}\,{{\rm{p}}{\rm{C}}{\rm{O}}}_{2}={{\rm{p}}{\rm{C}}{\rm{O}}}_{2}\times {\rm{E}}{\rm{X}}{\rm{P}}(0.0423\times ({{\rm{s}}{\rm{s}}{\rm{t}}}_{{\rm{m}}{\rm{e}}{\rm{a}}{\rm{n}}}-{\rm{s}}{\rm{s}}{\rm{t}}));$$

2 $${\rm{t}}{\rm{h}}{\rm{e}}{\rm{r}}{\rm{m}}{\rm{a}}{\rm{l}}\,{{\rm{p}}{\rm{C}}{\rm{O}}}_{2}={{\rm{p}}{\rm{C}}{\rm{O}}}_{2{\rm{m}}{\rm{e}}{\rm{a}}{\rm{n}}}\times {\rm{E}}{\rm{X}}{\rm{P}}(0.0423\times ({\rm{s}}{\rm{s}}{\rm{t}}-{{\rm{s}}{\rm{s}}{\rm{t}}}_{{\rm{m}}{\rm{e}}{\rm{a}}{\rm{n}}}));$$

where at each grid point, *pCO*_2_ is the oceanic pCO_2_ at a given point in time, *pCO*_*2 mean*_ is the mean pCO_2_ over the whole time period, *sst* is the SST at the given point in time, and *sst*_*mean*_ is the mean SST of the whole time period. Following Landschützer *et al*.^22^, we compute ΔpCO_2_ by subtracting the atmospheric pCO_2_ at each grid point from the oceanic pCO_2_ at the same grid point. We obtain atmospheric xCO_2_ from the NOAA marine boundary layer reference product (https://www.esrl.noaa.gov/gmd/ccgg/mbl/). From this, we calculate atmospheric pCO_2_ as outlined in Landschützer *et al*.^46^ using the NCEP sea level pressure^48^ and the water vapour correction by Dickson *et al*.^49^.

### Ocean sectors and position of fronts

To analyse sectoral differences within the Southern Ocean, we define the Atlantic sector from 70°W to 20°E, the Indian sector from 20°E to 145°E, and the Pacific sector from 145°E to 70°W (see Fig. 1A). We chose to divide the Southern Ocean into these sectors and not, e.g., into water masses, because the sectors are separated by fixed lines, while other ways of dividing the ocean are dynamic and not straight-forward. In addition, similar processes are at play within each of the sectors. Furthermore, several fronts separate the Southern Ocean and divide it into inter-frontal zones with unique biogeochemical and physical properties. For our analysis and discussion, we use the Subtropical Front (STF) at ~40°S and the Polar Front (PF) at ~55°S as defined by Orsi *et al*.^50^ (see Fig. 1A). Although we use the mean position of the fronts, the positions of the fronts are not static as they change their position on time scales from intra- to inter-annual^51^.

## Supplementary information

Supplementary Information

## Data Availability

The datasets generated during the current study are available from NOAA OCADS (https://www.nodc.noaa.gov/ocads/oceans/SPCO_2__1982_present_ETH_SOM_FFN.html). All remaining data analysed during this study are included in this published article (and its Supplementary Information files).
