# Supplementary material for: Regional Wind Variability Modulates the Southern Ocean Carbon Sink
Source: Sci Rep. 2019 May 14;9:7384. doi: 10.1038/s41598-019-43826-y (PMC6517586; doi:10.1038/s41598-019-43826-y)
Supplement: Supplementary file 1 — Supplementary Information [file 41598_2019_43826_MOESM1_ESM.pdf]

Supplementary Information for

Regional Wind Variability Modulates the Southern Ocean Carbon Sink

Lydia Keppler[1,2*] and Peter Landschützer[1]

Author affiliations

[1]The Max-Planck-Institute for Meteorology, Bundesstraße 53, 20146 Hamburg, Germany.

[2]International Max Planck Research School on Earth System Modelling (IMPRS-ESM), Bundesstraße 53, 20146 Hamburg, Germany

***Corresponding author**

Email: lydia.keppler@mpimet.mpg.de

This file includes:

- Supplementary text S1 to S7
- Figs. S1 to S7
- References for SI reference citations

Supplementary Information Text

S1. The effect of Sea Level Pressure on the Southern Annular Mode (SAM)

Previous studies have shown that the SAM has a zonal asymmetry that affects the mixed-layer depth and temperature[1,2]. Fig. S1 highlights the zonal asymmetry of the SAM, indicating that the negative correlation between the SAM and sea level pressure reaches further north in the eastern Pacific compared to the rest of the study region.

S2. The available carbon dioxide (CO_2) observations and robustness of the interpolated data

The Southern Ocean is historically an under-sampled region, due to the harsh conditions and the relative remoteness. The available data of the partial pressure of CO_2 (pCO_2) in the Southern Ocean come mainly from shipboard measurements, which have steadily increased in number in the past several decades, with the Drake Passage and the Tasman Sea being the best-sampled region within the Southern Ocean since the 2000s (Fig. S2*A*)[3,4]. Despite the substantial increase in observational pCO_2 data in the Southern Ocean, the spatio-temporal distribution remains sparse compared to other regions[3]. The robustness of the neural-network interpolated pCO_2 data until December 2011 has been demonstrated in previous studies[5–7]. Here, we show the robustness of the method for the most recent period.

Averaged over the most recent time period (2012 through 2016) the observations (Fig. S2*B*) are relatively well represented in the interpolated pCO_2 (Fig. S2*C*). Although some regional biases are present (Fig. S2*D*), they mostly cancel out when averaged over the study region (1.4 μatm). The standard deviation at each grid point is shown in Fig. S2*E*, which add up to 5.6 μatm. The Antarctic coastal areas display the largest standard deviation; however, our study mainly focuses on observations north of 65°S, due to the data availability of the temperature and salinity.

S3. The effect of the El Niño Southern Oscillation (ENSO) on the Southern Ocean air-sea CO_2 flux

We investigate the effect of the ENSO on the Southern Ocean carbon uptake, similarly as we did for the SAM (Fig. S3). We find the regional effect of the ENSO considerably smaller than the regional effect by the SAM. However, similar as the SAM, integrated over each of the three sectors, and over the whole Southern Ocean, the net effect of the ENSO on the Southern Ocean carbon uptake is ~zero.

S4. The regional relationship between sea surface temperature (SST) and the CO_2 flux

The air-sea CO_2 flux partially depends on SST, as CO_2 dissolves better in colder water However, other factors including biological production and vertical circulation also affect the oceanic CO_2 uptake. Here, we demonstrate the relationship between the SST and the air-sea CO_2 flux in the different sectors and interfrontal zones from 2004 through 2016 (Fig. S4).

In all three sectors of the Antarctic Zone, (AAZ, from 65°S to ~55°S), the SST ranges approximately from -2 to 5°C, and the carbon flux varies approximately from -5 to 3 mol m^{-2} $year^{-1}$. There is a slight trend where warmer surface waters tend to coincide with more uptake in the Atlantic and Indian sectors of the AAZ, indicating that solubility is not the main driver here. Concurrently, the Pacific sector of the AAZ does not show a considerable trend.

In the Polar Frontal Zone (PFZ, from ~55°S to ~40°S), the SST ranges approximately from 0 to 17°C, and the carbon flux varies from approximately -4 to 2 mol m^{-2} $year^{-1}$. In the Atlantic and Indian sectors of the PFZ, warmer surface waters tend to coincide with more uptake. However, the Pacific sector is a lot more variable and the colder surface waters have a similar

trend as the Atlantic and Indian sectors, but the warmer waters in this region have a reversal of this trend.

In the Subtropical Zone (STZ, from ~40°S to 30°S), the SST ranges approximately from 10 to 25°C, and the carbon flux varies approximately from -5 to 1 mol m^{-2} $year^{-1}$. In all three sectors of the STZ, there is a trend of colder surface waters tending to coincide with more uptake.

S5. Mean sea surface properties and air-sea CO_2 flux of the sectors

Here, we show the zonal mean sea SST, sea surface salinity (SSS) and the air-sea CO_2 flux in the Southern Ocean sectors to provide context (Fig. S5); the anomalies are shown in S6.

S6. Sea surface property anomalies and the air-sea CO_2 flux anomalies of the sectors

We analyse the sea-air CO_2 flux anomalies in comparison with the physical sea surface properties of the Southern Ocean (Fig. S6). Generally, we find that warmer and saltier surface waters tend to coincide with a stronger carbon sink. Therefore, it is evident that solubility is not the dominant mechanism driving the sink variability, but rather reflects the reduction in vertical mixing that usually brings cool but carbon-rich deep water to the surface. However, strong differences exist between both the different sectors and the interfrontal zones (see also S4).

Similar to the sea-air CO_2 flux, the SST and SSS anomalies are strongest in the Atlantic sector (Fig. S6*A*-*C*). Here, the temporal evolution is dominated by a warm anomaly in 2011, north of the Polar Front (PF), which coincides with saltier surface waters and a stronger carbon sink. A similar, but weaker anomalous period can be observed around 2009, north of the PF. After this event, the Atlantic sector becomes cooler and fresher, while the carbon sink becomes weaker again, especially north of the PF.

In the Pacific sector, the signal varies on shorter time scales compared to the Atlantic sector in all three variables (Fig. S6*D-F*), which strongly coincides with the ENSO (see S3), suggesting the influence of remote modes of variability on the Southern Ocean CO_2 sink. While throughout the time period, warm SST anomalies usually coincide with saltier phases and vice versa, the carbon sink appears disconnected from this pattern. Warmer and saltier waters coincide with less carbon uptake in the PFZ from 2004 through 2011, which was previously[7] considered as evidence that the CO_2 flux trends in the Pacific sector are solubility dominated[8]. However, subsequently colder and fresher waters coincide with less carbon uptake in the PFZ after 2011, challenging this view.

The Indian sector behaves similarly to the Atlantic sector in terms of SST, similar to the Pacific sector in terms of SSS, and like a mixture between the Atlantic and Pacific sectors in terms of its CO_2-flux (Fig. S6*G-I*). For example, the period of much higher SST around 2011 north of the PF that was observed in the Atlantic, is also present in the Indian sector, albeit with less intensity. In the following years, the surface waters in the STZ of both the Pacific and the Indian sectors are saltier than the mean. This trend moves further south in ~2016.

S7. Trends of the pCO_2, its components, and drivers in the reinvigoration period

Here, we show the trends of the reinvigoration period to put our findings on the trends during the most recent period in context. As discussed in Landschützer et al. [2015][8], in the reinvigoration period, the ΔpCO_2 decreased in the Southern Ocean (Fig. S7*A*), resulting in enhanced CO_2 uptake by the ocean. The authors demonstrated that in this period the westerly winds were stronger in the Pacific and weaker in the Atlantic due to a dipole in sea level pressure (Fig. S7*D*). This change in surface wind patterns is thought to have caused enhanced downwelling and warmer surface waters in the Atlantic sector. The non-thermal component (Fig. S7*C*) dominated over the thermal component (Fig. S7*B*), resulting in an overall decrease in

ΔpCO_2 (Fig. S7*A*). Concurrently, the stronger westerlies in the Pacific sector caused enhanced upwelling and colder surface waters. Here, the thermal component (Fig. S7*B*) dominated over the non-thermal component (Fig. S7*C*), resulting in an enhanced CO_2 uptake by the ocean.

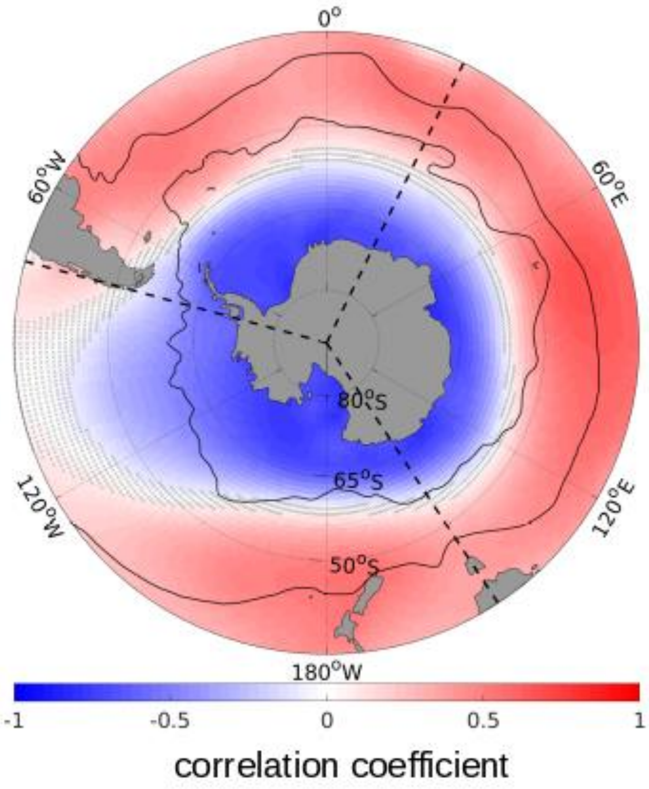

Fig. S1. As Fig. 2*B* but with the correlation between the SAM and sea level pressure: Correlation coefficients between the sea level pressure [hPa] and the standardized SAM index, smoothed with a 3-month running average, between January 1982 and December 2016. Coefficients with significance <95% are hatched. The mean positions of the PF (~55°S) and the STF (~40°S) are illustrated as thin black lines, and the three Southern Ocean sectors are delimited by dashed black lines.

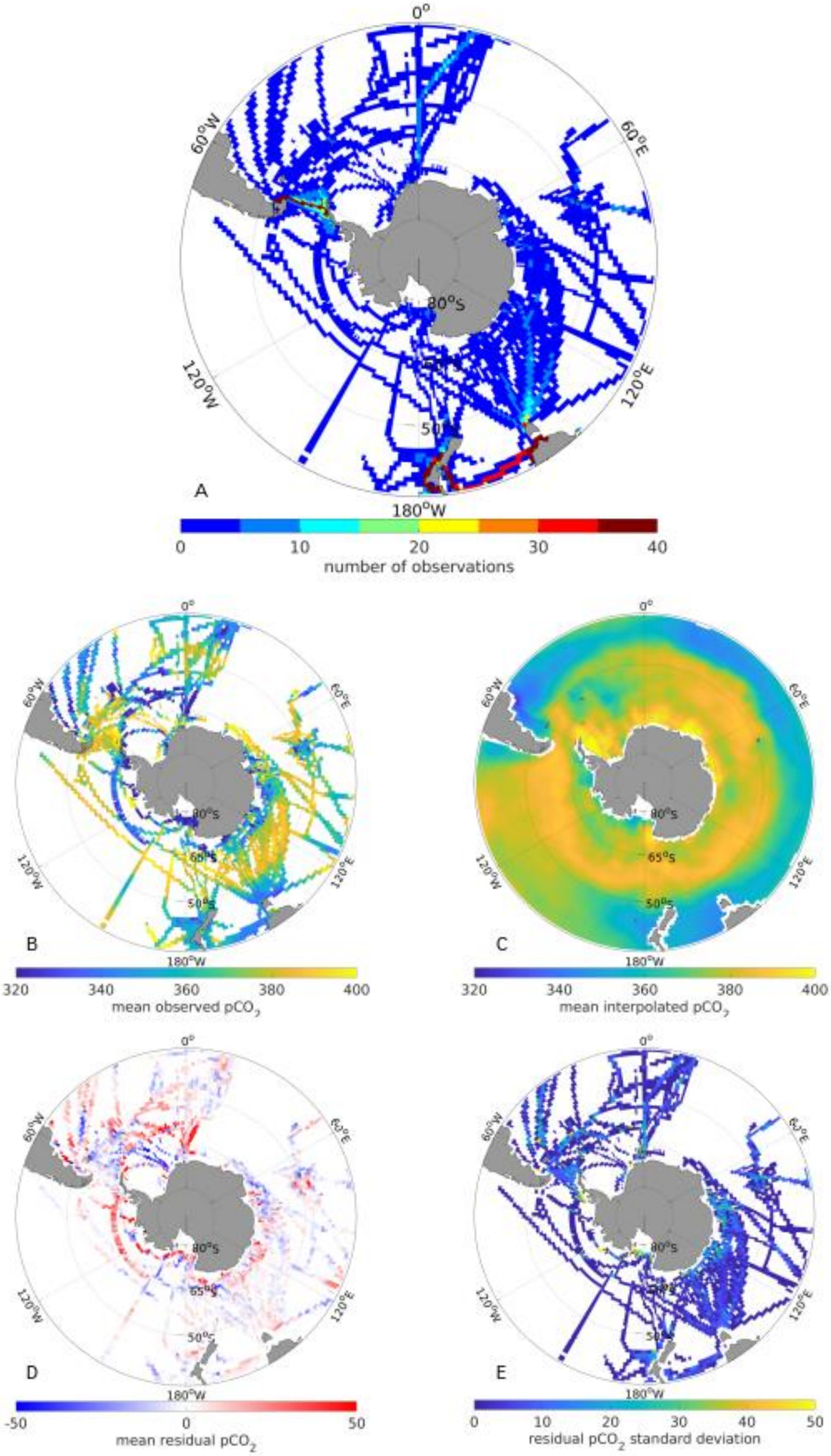
0°
60°W
60°E
80°S
120°W
120°E
50°S
A
180°W
0
10
20
30
40
number of observations
B
180°W
320
340
360
380
400
mean observed pCO2
C
mean interpolated pCO2
65°S
D
-50
0
50
mean residual pCO2
E
residual pCO2 standard deviation

Fig. S2. Available pCO_2 observations [μatm] and robustness of the interpolated data in the most recent period (2012 through 2016): *(A)* The distribution of the shipboard pCO_2 observations in the Southern Ocean from the SOCATv5 database for each 1°x1° grid point *(B)* The mean observed pCO_2 from the SOCATv5 database *(C)* The mean interpolated pCO_2 from Landschützer et al. [2015] *(D)* The mean residual pCO_2 (interpolated minus observed pCO_2). *(E)* The standard deviation of the residual pCO_2.

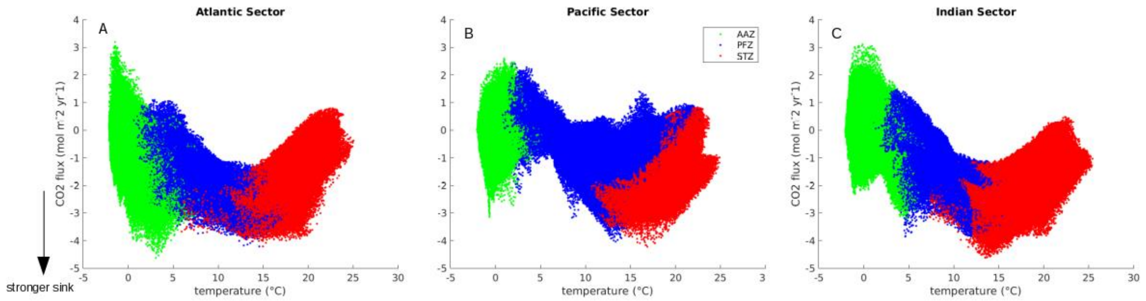

Fig. S3. CO_2 flux as a function of SST in each of the Southern Ocean sectors and interfrontal zones, using monthly means from 2004 through 2016: Atlantic *(A)*, Pacific *(B)*, and Indian sector *(C)*, for the AAZ (green), PFZ (blue), and STZ (red).

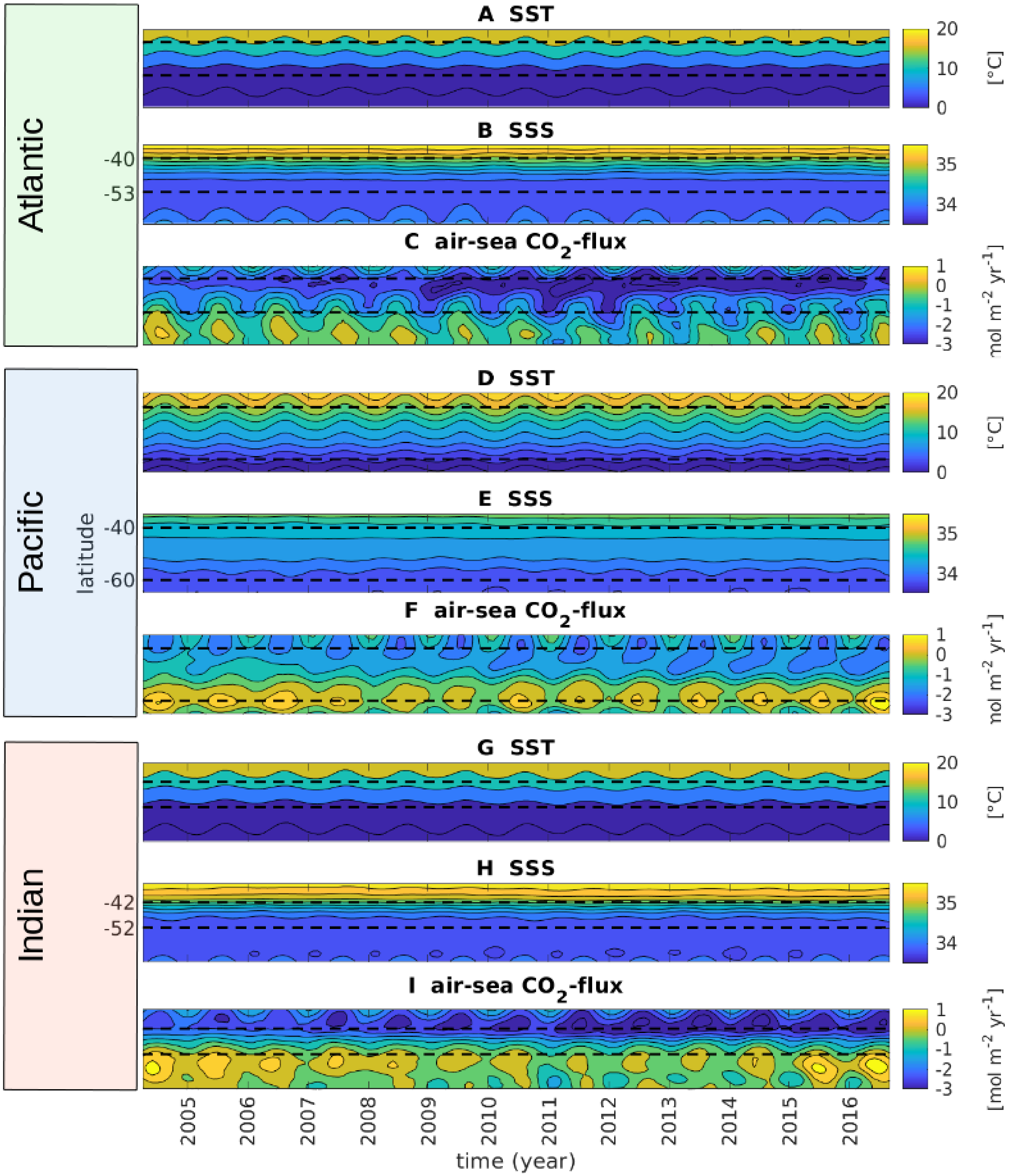

Fig. S4. As Fig. 2, but the mean instead of the anomalies: Hovmöller plots of the zonal means of the Southern Ocean sectors as a function of time (x-axis) and latitude (y-axis) from 35°S to 65°S. SST [°C] *(A,D,G)* and SSS *(B,E,H)* in comparison to the carbon flux [mol m-^{2} yr^{-1}] *(C,F,I)* for the Atlantic *(A-C)*, Pacific *(D-F)*, and Indian sectors *(G-I)*. The mean positions of the STF (40°S, 40°S, 42°S) and PF (53°S, 60°S, 52°S) are shown for the Atlantic, Pacific, and Indian sectors respectively as dashed black lines. Negative values in the CO_2-flux indicate oceanic uptake. The seasonal cycle is not removed, but we smoothed with a 3-month running mean.

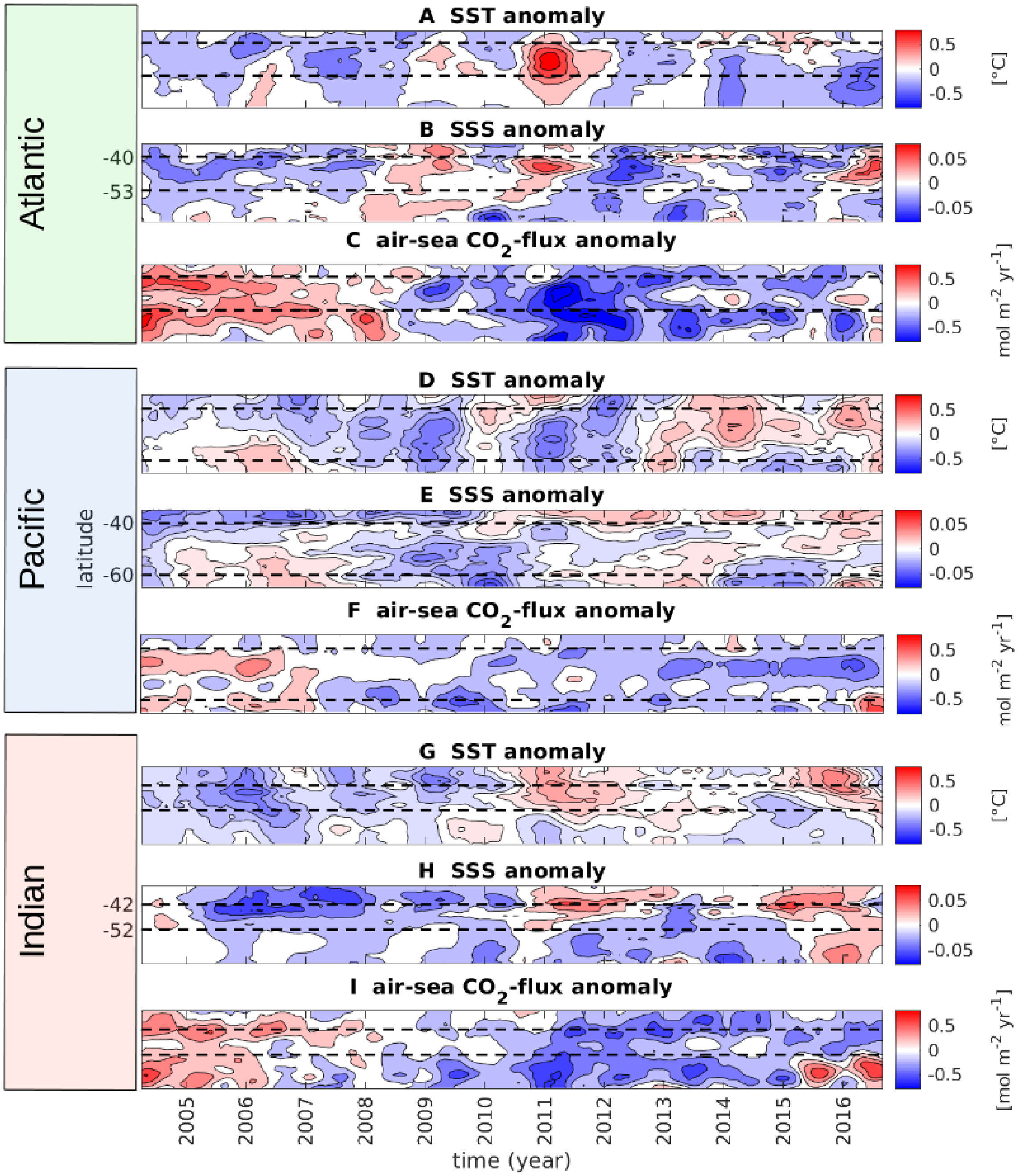

Fig. S5. Hovmöller plots of the zonal mean anomalies of the Southern Ocean sectors as a function of time (x-axis) and latitude (y-axis) from 35°S to 65°S. SST anomalies [°C] *(A,D,G)*, SSS anomalies *(B,E,H)* and the carbon flux anomalies [mol m^{-2} yr^{-1}] *(C,F,I)* for the Atlantic *(A-C)*, Pacific *(D-F)*, and Indian sectors *(G-I)*. The mean positions of the Subtropical Front (STF, 40°S, 40°S, 42°S) and PF (53°S, 60°S, 52°S) are illustrated for the Atlantic, Pacific, and Indian sectors respectively as dashed black lines. The anomalies are based on the mean between 2004 and 2016, and the first and last 3 months are removed in the smoothing. Negative values in the CO_2-flux anomalies indicate a stronger sink. Note that while Fig. 1 extends until the Antarctic coast (~77°S), Fig. 2 only extends until 65°S due to the data availability of the SST and SSS. See also S5 for the mean values instead of the anomalies.

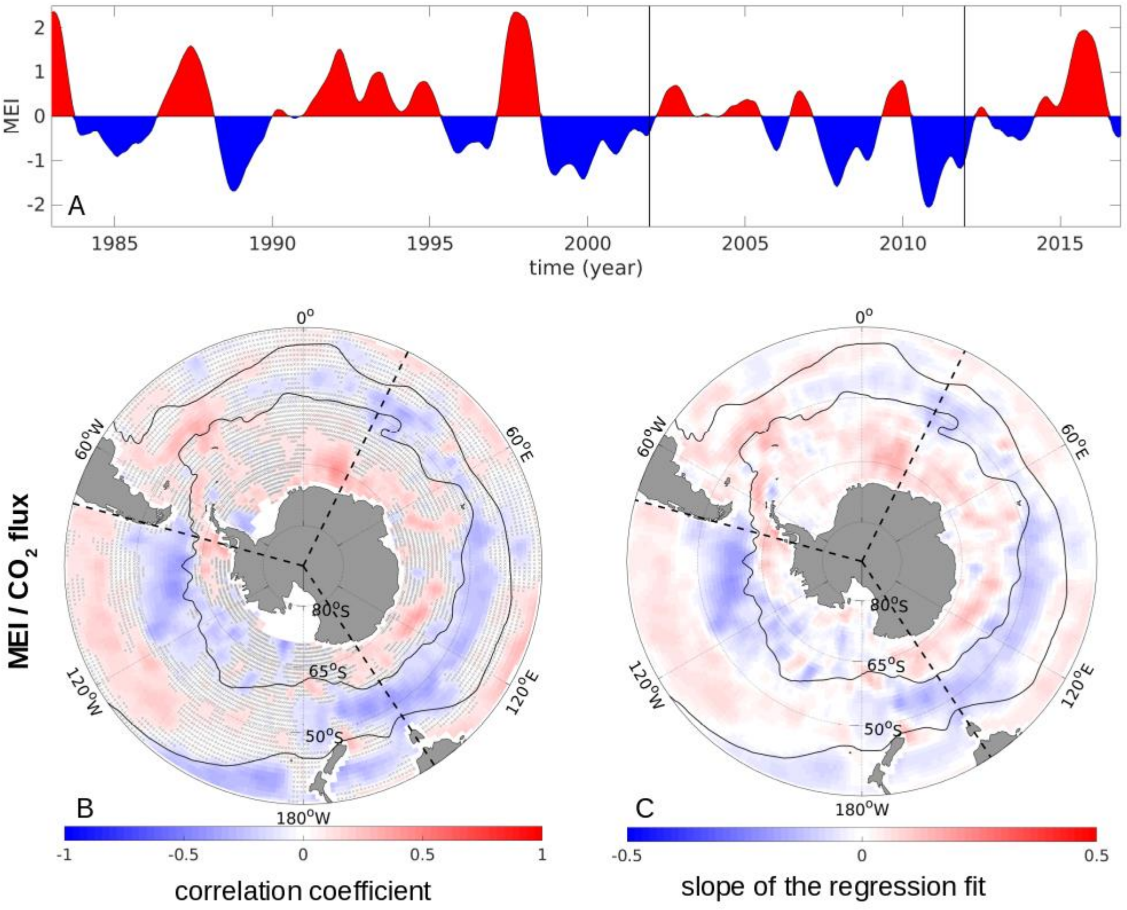

Fig. S6. As Fig. 4, but with the Multivariate ENSO Index (MEI) instead of the SAM index: The relationship between the MEI and the CO_2 flux anomaly between January 1982 and December 2016. *(A)* Standardized MEI smoothed with a 3-month running mean. Positive is shown in red, negative in blue. The start of the reinvigoration (Jan 2002) and the current period (Jan 2012) are marked with thin black lines. *(B)* Correlation coefficients between the air-sea CO_2 flux anomaly [mol m^{-2} yr^{-1}] and the standardized MEI. Coefficients with significance <95% are hatched. *(C)* The slope of the regression fit between the air-sea CO_2-flux anomalies [mol m^{-2} yr^{-1}] and the standardized MEI. As the MEI is standardized to have a mean of 0 and a standard deviation of 1, *(C)* illustrates the change in the CO_2 flux [mol m^{-2} yr^{-1}] per standard deviation of the MEI. *(B-C)* The mean positions of the PF (~55°S) and the STF (~40°S) are shown as thin black lines, the three Southern Ocean sectors are delimited by dashed black lines, and the coastal areas are masked white.

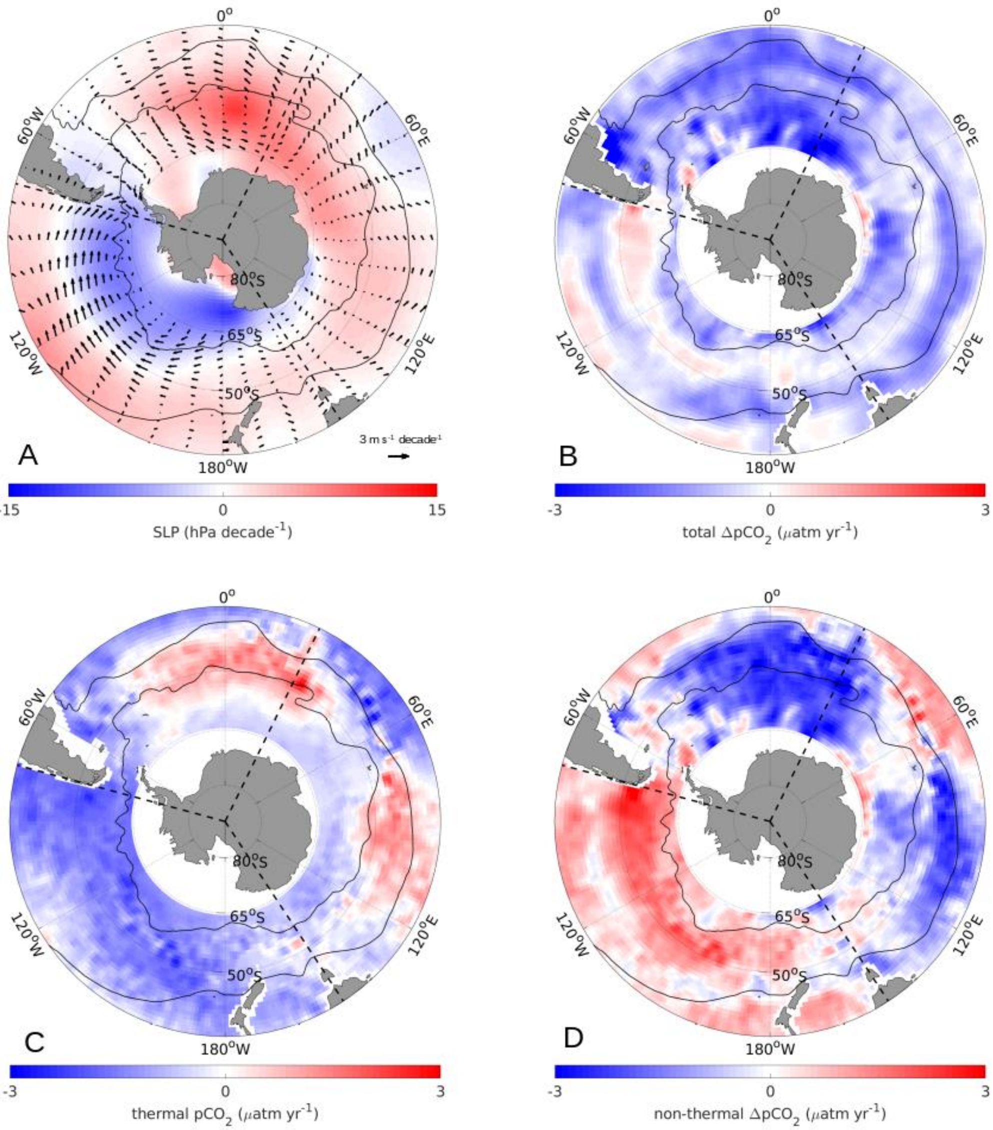

Fig. S7. As Fig. 4, but for the reinvigoration period (2004 through 2011) instead of the most recent period (2012 through 2016). Trends of the pCO_2, its components, and the sea level pressure and 10 m wind velocity during the reinvigoration period (2004 through 2011). *(A)* trend of the sea level pressure (hPa decade^{-1}) (color) and trend of the 10 m wind velocity (m s^{-1} decade^{-1}) (vectors). (*B*) Trend of the ΔpCO_2 (μatm year^{-1}); *(C)* trend of the thermal component of the pCO_2 (μatm year^{-1}); *(D)* trend of the non-thermal component of the ΔpCO_2 (μatm year^{-1}); The mean positions of the PF and the STF are shown as thin black lines and dashed black lines delimit the three Southern Ocean sectors. Note: a similar figure was shown in Landschützer et al. [2015] [8] for the period from 2002 through 2011. Note that the scale is smaller than in Fig. S3 for *B*-*D*.

References

1. Sallée, J. B., Speer, K. G. & Rintoul, S. R. Zonally asymmetric response of the Southern Ocean mixed-layer depth to the Southern Annular Mode. *Nat. Geosci.* **3**, 273–279 (2010).
2. Fogt, R. L., Jones, J. M. & Renwick, J. Seasonal Zonal Asymmetries in the Southern Annular Mode and Their Impact on Regional Temperature Anomalies. *J. Clim.* **25**, 6253–6270 (2012).
3. Bakker, D. C. E. *et al.* A multi-decade record of high-quality fCO2 data in version 3 of the Surface Ocean CO2 Atlas (SOCAT). *Earth Syst. Sci. Data* **8**, 383–413 (2016).
4. Munro, D. R. *et al.* Recent evidence for a strengthening CO2 sink in the Southern Ocean from carbonate system measurements in the Drake Passage (2002-2015). *Geophys. Res. Lett.* **42**, 7623–7630 (2015).
5. Landschützer, P. *et al.* A neural network-based estimate of the seasonal to inter-annual variability of the Atlantic Ocean carbon sink. *Biogeosciences* **10**, 7793–7815 (2013).
6. Landschützer, P., Gruber, N., Bakker, D. C. E. & Schuster, U. Recent variability of the global ocean carbon sink. *Glob. Biogeochem. Cycles* **28**, 927–949 (2014).
7. Landschützer, P., Gruber, N. & Bakker, D. C. E. Decadal variations and trends of the global ocean carbon sink. *Glob. Biogeochem. Cycles* **30**, 1396–1417 (2016).
8. Landschützer, P. *et al.* The reinvigoration of the Southern Ocean carbon sink. *Science* **349**, 1221–1224 (2015).
